# Population structure and residency patterns of whale sharks, *Rhincodon typus*, at a provisioning site in Cebu, Philippines

**DOI:** 10.7717/peerj.543

**Published:** 2014-09-16

**Authors:** Gonzalo Araujo, Anna Lucey, Jessica Labaja, Catherine Lee So, Sally Snow, Alessandro Ponzo

**Affiliations:** 1 Large Marine Vertebrates Research Institute Philippines, Jagna, Bohol, Philippines; 2 Physalus NGO, Large Marine Vertebrates Project Philippines, Largo Callifonte, Rome, Italy

**Keywords:** Residency, Lagged identification rate, Whale shark, Population, Oslob, Provisioning, Philippines

## Abstract

This study represents the first description of whale sharks, *Rhincodon typus*, occurring at a provisioning site in Oslob, Cebu, Philippines. Frequent observations of sharks are often difficult, even at tourism sites, giving rise to provisioning activities to attract them. The present study provides repeated longitudinal data at a site where daily provisioning activities took place, and whale sharks were present every day. A total of 158 individual whale sharks were photographically identified between Mar 2012 and Dec 2013, with 129 males (82%), 19 females (12%) and 10 (6%) of undetermined sex. Mean estimated total length was 5.5 m (±1.3 m S.D.). Twenty individuals were measured with laser photogrammetry to validate researchers’ estimated sizes, yielding a good correlation (*r*^2^ = 0.83). Fifty-four (34%) individuals were observed being hand-fed by local fishermen (provisioned), through in-water behavioural observations. Maximum likelihood methods were used to model mean residency time of 44.9 days (±20.6 days S.E.) for provisioned *R. typus* contrasting with 22.4 days (±8.9 days S.E.) for non-provisioned individuals. Propeller scars were observed in 47% of the animals. A mean of 12.7 (±4.3 S.D.) *R. typus* were present in the survey area daily, with a maximum of 26 individuals (Aug 10 2013) and a minimum of 2 (Dec 6 2012). Twelve (8%) individuals were seen on at least 50% of survey days (*n* = 621), with a maximum residency of 572 days for one individual (P-396). Twenty four individuals were photographically identified across regional hotsposts, highlighting the species’ migratory nature and distribution. Extended residency and differences in lagged identification rates suggest behavioural modification on provisioned individuals, underlying the necessity for proper management of this tourism activity.

## Introduction

Provisioning food is a means of attracting wildlife to facilitate human interaction, and though it is a widespread practice, its long-term ecological implications need further investigation (Orams, 2002; Dobson, 2006). Reliable shark encounters are difficult, promoting the use of provisioning activities to attract them (Gallagher & Hammerschlag, 2011; Hammerschlag et al., 2012) These are controversial as sharks are apex predators, and some provisioned species are potentially dangerous to humans and may impact their ecological function (Brunnschweiler & McKenzie, 2010). In the Red Sea, tagged silky sharks, *Carcharhinus falciformis*, had modified local habitat use and increased presence on days when baiting occurred (Clarke, Lea & Ormond, 2011). Similarly, studies on sicklefin lemon sharks, *Negaprion acutidens*, in French Polynesia, showed an increase in residency and abundance over time, as well as modified intraspecific behaviour resulting from an increase in dominance actions and aggression to acquire food (Clua et al., 2010). Though increased residency can have a negative effect on gene flow, and lead to reproductive isolation over time, there is a lack of baseline data at study sites for comparison (Clua et al., 2010). Whitetip reef sharks, *Triaenodon obesus*, in Australia, showed different daily activity, as measured by vertical movement with temperature-depth-recorder tags, when provisioning took place in the area (Fitzpatrick et al., 2011). In contrast, separate studies on tiger sharks, *Galeocerdo cuvier*, and Caribbean reef sharks, *Carcharhinus perezi*, at provisioning sites exhibited no activity space restriction and no significant difference in residency compared with non-provisioned populations, respectively (Maljković & Côté, 2011; Hammerschlag et al., 2012). The economic importance of tourism led by provisioning interactions with elasmobranchs is substantial (Clua et al., 2011; Rowat & Engelhardt, 2007; Topelko & Dearden, 2005; Gallagher & Hammerschlag, 2011). However, to fully grasp the ecological impact of such activities, longitudinal long-term monitoring research is necessary on its adjacent communities and environments, as suggested by Brunnschweiler, Abrantes & Barnett (2014).

The whale shark, *Rhincodon typus*, is known to inhabit tropical and subtropical waters, and aggregate predictably in several hotspots around the world, which has been primarily linked to high productivity areas (Colman, 1997b; Eckert et al., 2002; Graham & Roberts, 2007; Martin, 2007; Nelson & Eckert, 2007; Rowat et al., 2007; de la Parra Venegas et al., 2011; Rowat & Brooks, 2012; Fox et al., 2013). Their diet consists primarily of surface zooplankton, though recent evidence suggests whale sharks are also feeding on demersal macroplankton and deep-water fishes (Rohner et al., 2013).

The predictability of their occurrence at these hotspots has led to the development of large tourism industries around these aggregations (Davis et al., 1997; Graham, 2007; Catlin & Jones, 2010; Gallagher & Hammerschlag, 2011). Though many are advertised as ecotours, the widespread use of the term has led to a loss of definition (Honey, 2008). Poor and unregulated whale shark tourism can lead to short and potentially long-term impacts, like behavioural change and displacement from critical habitats (Norman, 2002; Quiros, 2007; Remolina-Suárez et al., 2007). Most whale shark aggregations are located in developing or newly industrialised countries, making the management of this resource a greater challenge (Rowat & Brooks, 2012).

The use of photographic identification (Photo-ID) in elasmobranchs is a reliable, minimally invasive means of obtaining population information, when its assumptions are met (Marshall & Pierce, 2012). By utilising the unique spot pattern present on the body of *R. typus*, individuals can be identified, and thus their residency and movements can be studied (Arzoumanian, Holmberg & Norman, 2005; Brooks et al., 2010). The citizen science contributing to ‘Wildbook for Whale Sharks’ (www.whaleshark.org) can help match individual *R. typus* between areas by allowing members of the public to submit photographs of the animals and encounter details. Opportunistic Photo-ID of individual *R. typus* can work as photographic mark-recapture data against modified maximum likelihood models to understand their residency and movement patterns (Whitehead, 2001; Wimmer & Whitehead, 2005; Fox et al., 2013).

Whale sharks inhabit the seas around the Philippine archipelago, with the most famous aggregation occurring in the waters of Donsol, Sorsogon Province (Eckert et al., 2002; Quiros, 2005; Pine, Alava & Yaptinchay, 2007; Quiros, 2007). When the aggregation was first identified in 1997, it attracted tourists and hunters alike, leading to the fishing of seven *R. typus*, followed by public outcry and campaigning across the country to protect the species. This successfully resulted in the passing of a national law protecting the whale shark from consumptive use and exploitation (FAO 193, Department of Agriculture, Quezon City, Philippines, March 25th 1998). Supported by WWF-Philippines, the fishing town of Donsol developed into the first whale shark tourism destination in the country (Quiros, 2005; Pine, Alava & Yaptinchay, 2007). The only other known aggregation of *R. typus* in the Philippines, was identified by O’Farrell et al. (2006) off Panaon Island, Southern Leyte. This was further described by Quiros et al. (2007), where a total of 62 encounters with 28 individual whale sharks were recorded over seven days.

In the Municipality of Oslob, located on the south of Cebu Island in the Central Visayas region of the Philippines, whale shark hunting was never confirmed (Alava et al., 2002). However, in nearby areas of the Bohol Sea, nearly 700 *R. typus* were landed at two monitoring sites, between 1993 and 1997 (Alava et al., 2002). The present study is the only detailed description of whale sharks presence in the Municipality of Oslob and examines the population structure and residency patterns of individuals identified at this provisioning site. Data from daily photographic identification was used to compare against residency models, using maximum likelihood methods (Whitehead, 2001). These methods were previously applied on other whale shark aggregations (Ramírez-Macías et al., 2012; Ramírez-Macías, Vázquez-Haikin & Vázquez-Juárez, 2012; Fox et al., 2013) as they use identification data to establish the spatial and temporal distribution of effort (Whitehead, 2001).

## Materials & Methods

All the methods here presented were conducted in accordance with national and local laws in respect of animal welfare. The Bureau of Fisheries and Aquatic Resources—Region 7, issued the authors a Gratuitous Permit, and a Memorandum of Agreement was signed with the Department of Environment and Natural Resources and DA-BFAR7. The Municipality of Oslob granted the authors a Prior Informed Consent document.

### Study site

**Figure 1:**
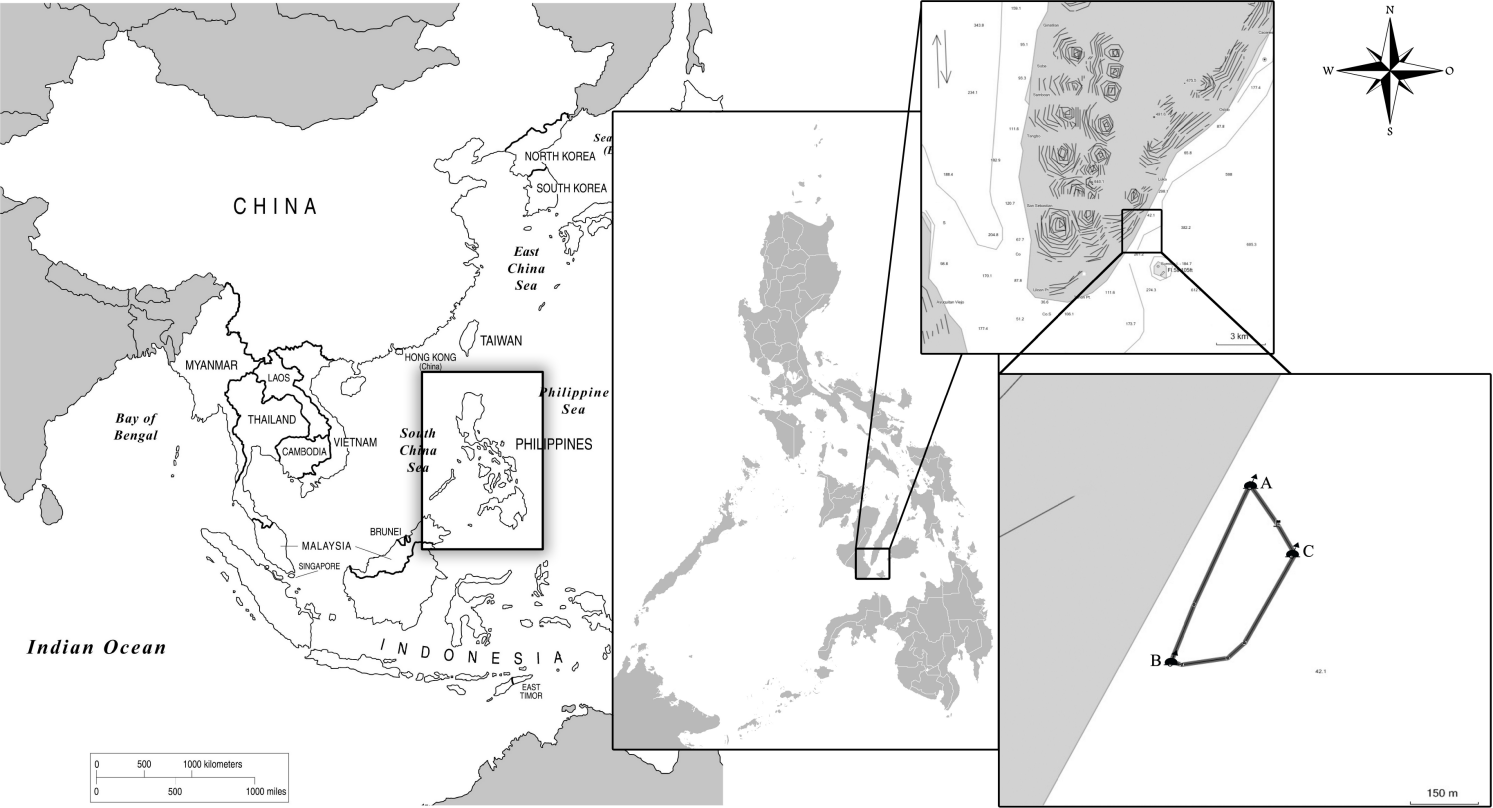
Map of the study site and interaction area demarked by buoys (A, B, C) in Barangay Tan-Awan, Municipality of Oslob, Cebu Province, Philippines.

Small buoys connected by a floating line demark the interaction area in the waters of Barangay (village) Tan-Awan, inside which only accredited non-motorised vessels are allowed. Fifty to 100 m offshore within the interaction area, feeders belonging to the TOSWFA people’s organisation are allowed by the municipal government to provision *R. typus* from one-man paddleboats. Provisioning takes place between 6 am and 1 pm, with 50–150 kg of food utilized for the provisioning daily, depending on the number of sharks and tourists present. The interaction area is semi circular, measuring 480 m from buoy “A” (N9 27 48.6 E123 22 48.4) to buoy “B” (N9 27 34.1 E123 22 43.0), and 170 m at its widest point to buoy “C” (N9 27 42.7 E123 22 52.7), for a total surface area of 65,457 m^2^ (Fig. 1). Researchers snorkelled out from shore and surveyed the area for presence of whale sharks. All recorded encounters with *R. typus* occurred within the demarked area. Systematic data collection described in this study took place between March 31st 2012 and December 31st 2013.

### Photographic identification

Photo-identification was conducted three times a day, between 7–8 am, 9–10 am and 11–12 pm. These three daily sessions were carried out through the whole study period, conditions and weather permitting. Photo-ID was used to describe the population as a non-invasive means to gather size, sex, and presence information of the animals. Over 350,000 photographs were taken and analysed for the present study. Upon sight of an *R. typus* in the water, researchers would first approach the left side of the animal, position themselves perpendicular to the animal and take a photograph of the unique spot pattern behind the gill slits above the pectoral fin (Arzoumanian, Holmberg & Norman, 2005). Upon a successful left-side view photograph of the animal, the researcher swam under the animal in order to identify the presence or absence of claspers. Sex was attributed to an individual if a cloaca region photograph was collected. The researcher would then capture the right-hand spot pattern of the animal as with the left one. Additional photographs were taken to capture any scars, abrasions, lacerations and any body truncations. Observations on the feeding behaviour were also noted on individuals. Though the whole population is possibly provisioned, as attracted to the study site by the presence of food (Orams, 2002), for the purpose of this study *R. typus* were considered “provisioned” when they were directly observed being hand-fed from a feeder boat. Caution should be taken as these direct observations on individuals cannot cover the whole of the animal’s time at the study site, and it is therefore possible that some non-provisioned individuals did indeed feed from feeder boats. Differences in size, sex, scarring, and presence were investigated amongst the two groups. The size of whale sharks was estimated by photographing the animal next to snorkelers or boats of known size. Pearson’s chi-squared test was applied to evaluate any bias amongst sex-identified *R. typus* from an assumed 1:1 female to male ratio (Joung et al., 1996). Wilcoxon–Mann–Whitney (WMW) tests were run amongst results to test for significance (Fay & Proschan, 2010). Statistical significance was tested at *P* = 0.05. Linear regression models were used to test relationship between variables. All statistical analyses were run on R version 3.0.1 (http://www.R-project.org).

The photographer visually identified each photographed individual. Two experienced researchers gave further validation to the identity of the individual before being inputted into a local I^3^S catalogue (http://www.reijns.com/i3s) (Van Tienhoven et al., 2007). Sex, estimated size and presence or absence of scars was also noted and inputted into the database. New, previously unidentified individuals were also uploaded to the online whale shark database “Wildbook for Whale Sharks” at www.whaleshark.org. The daily presence of every *R. typus* was recorded on a spreadsheet. Celestial information, defined by moon irradiance (www.timeanddate.com; accessed on January 4th 2014), was investigated through linear regression as a possible variable affecting variation in daily presence of *R. typus* individuals in the study area (Graham, Roberts & Smart, 2006).

### Photogrammetry

Size of *R. typus* was estimated by photographing the animals next to snorkelers and/or boats of known size parallel to the animals. Additionally, laser photogrammetry was used to validate researchers’ estimated total lengths. Parallel green lasers (Sea Turtle Scuba Inc.; http://www.seaturtlescuba.us/) were placed on individual arms at the extremities of a custom-made frame 30 cm apart, with an underwater camera placed in the centre of the two. Parallel alignment of the lasers was verified before and after each in-water session by measuring the distance between the two projected laser dots on a parallel surface placed at 1 and 5 m away. During each dedicated 30-min measuring session, or otherwise limited by battery life, the researcher swam in the interaction area measuring free-swimming *R. typus*. Only pictures taken perpendicularly to the animal (90°) were used for analysis. Poor visibility during the study period and the limited field-of-view of the camera used with the photogrammetry set-up, made it difficult to obtain a clear photograph of the entire length of a whale shark, therefore Rohner et al. (2011) methods were used to estimate total length. In the aforementioned study, the distance between the fifth gill slit and a perpendicular line to the body axis passing on the anterior margin of the first dorsal fin is used in relation to the total length of animal, measured from free-swimming and stranded animals, found to be *y* = 4.8373*x* + 80.994 (*r*^2^ = 0.93). A total of 190 photograph measurements were used for analysis on 20 individuals and fitted to the abovementioned equation.

### Residency and lagged identification rate

Maximum likelihood methods were used to model and estimate overall residency time of *R. typus* in the study area. Program SOCPROG 2.4 (Whitehead, 2009) was used to calculate the lagged identification rate (LIR), which is the probability that an identified animal will be resighted in the study area after a certain time period (Whitehead, 2001). Eight models (Table 1) were compared to the empirical data, and tested for goodness of fit, with days as units and no group variables included in the “Movement” module of the software. The quasi-Akaike information criterion (QAIC) was used to evaluate each model’s results and account for over-dispersion of data, and the summed log likelihood (SLL) was used as a performance evaluator of each model (Whitehead, 2007). With the dataset in its entirety, the best-fitting model was a poor approximation of the empirical results, with LIR tapering to zero after 500–600 days. The empirical data clearly showed that individuals who proactively fed from provisioning boats had a longer residency pattern to those who didn’t, causing large over-dispersion of the data. The data was therefore separated to run the models independently. The criterion for separation was “feeding behaviour”, where one dataset would contain *R. typus* who had been observed feeding from feeder boats (*n* = 54) defined as provisioned, and *R. typus* who had been identified but never observed feeding from feeder boats (*n* = 104), defined as non-provisioned. This was inputted as supplemental data into SOCPROG. Both datasets were run as described above, and results were found to better represent the data. The best-fit model (Model H, Table 1) for both provisioned and non-provisioned sharks, respectively, were bootstrapped for 100 repetitions, to estimate standard errors and provide 95% confidence intervals (Buckland & Garthwaite, 1991).

**Table 1:** Model parameters and comparison for lagged identification rate of *R. typus* at Oslob. Parameters as preset by Whitehead (2009) in SOCPROG 2.4. These parameters test from closed population models (A & B) to various combinations of emigration, reimmigration and mortality (C–H). The values displayed show the difference between the QAIC (quasi-Akaike information criterion) results obtained for each model and the smallest QAIC result.

Name	Model parameters	QAIC results: provisioned individuals	QAIC results: nonprovisioned individuals
A	Closed (1/*a*1 = *N*)	1484.97	927.50
B	Closed (*a*1 = *N*)	1484.97	927.50
C	Emigration/mortality (*a*1 = emigration rate; 1/*a*2 = *N*)	553.00	235.40
D	Emigration + reimmigration (*a*1 = emigration rate; *a*2/(*a*2 + *a*3) = proportion of population in study area at any time)	35.07	48.79
E	Emigration/mortality (*a*1 = *N*; *a*2 = mean residence time)	553.00	235.40
F	Emigration + reimmigration + mortality	493.93	185.20
G	Emigration + reimmigration (*a*1 = *N*; *a*2 = mean time in study area; *a*3 = mean time out of study area)	35.07	48.79
H	Emigration + reimmigration + mortality (*a*1 = *N*; *a*2 = mean time in study area; *a*3 = mean time out of study area; *a*4 = mortality rate)	0.00	0.00

**Notes.**

Where *N* is the population size in the study area; QAIC, quasi-Akaike information criterion.

## Results

### Population structure

**Figure 2:**
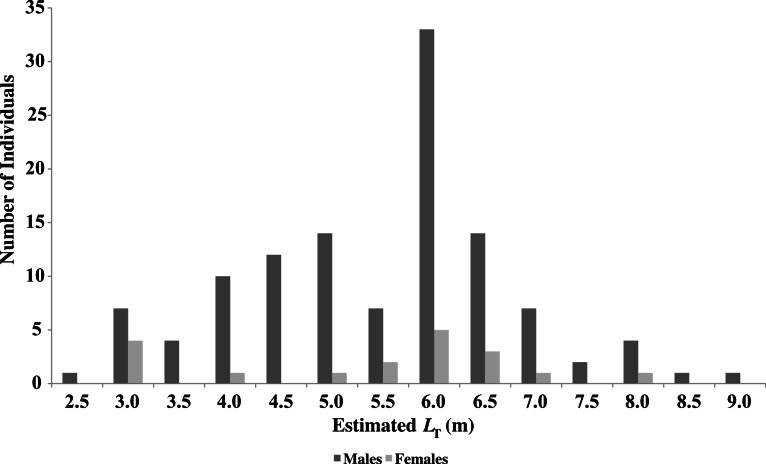
Sex and size distribution of *R. typus* identified in Oslob.

In 621 days of survey, during the 641 days included in the study period, 158 individual *R. typus* were identified. Sex was identified for 148 animals (94%), of which 129 were male (82%) and 19 were female (12%), highlighting a significantly male biased population (*χ*^2^ = 45.7, *P* = 1.37e−11). The estimated total length (*L_T_*) of the males (*n* = 118) had a mean value of 5.5 m (±1.3 m S.D.), whereas the females (*n* = 19) had a mean value of 5.4 m (±1.5 m S.D.) showing no significance in size distribution (*WMW*, *P* = 0.8722). Of the 129 males identified, 19 (14%) of them had claspers extending over the pelvic fins, with a mean estimated *L_T_* of 6.9 m (±1.1 m S.D., *n* = 18), considerably larger than the overall male mean estimated *L_T_* (*WMW*, *P* = 7.99^e−05^). Only four males were considered to be sexually mature based on the calcified appearance of the claspers and a mean *L_T_* over 8 m (Colman, 1997a). It was not possible to determine maturity of females and none were visibly pregnant. The mean size of the population was 5.5 m (±1.3 m S.D., *n* = 141), ranging from 2.5 m to 9 m (Fig. 2).

A total of 190 pictures were selected for photogrammetry measurement based on photograph quality. Twenty individual sharks were measured between the 5th gill slit and the start of the dorsal fin (*B*_*P*1_), yielding a mean total length based on *B*_*P*1_ measurements of 5.6 m (±0.7 m S.D.). The researchers’ visually estimated *L_T_* for these 20 individuals had a mean of 5.4 m (±1.3 m S.D.), a small difference of 0.2 m (±0.7 m S.D., *WMW*, *P* = 0.4885). Linear regression of the researchers’ estimated *L_T_* and the results from the *B*_P1_*L_T_*, yielded a significant relationship (*r*^2^ = 0.83, *P* = 2.57^e−08^).

**Figure 3:**
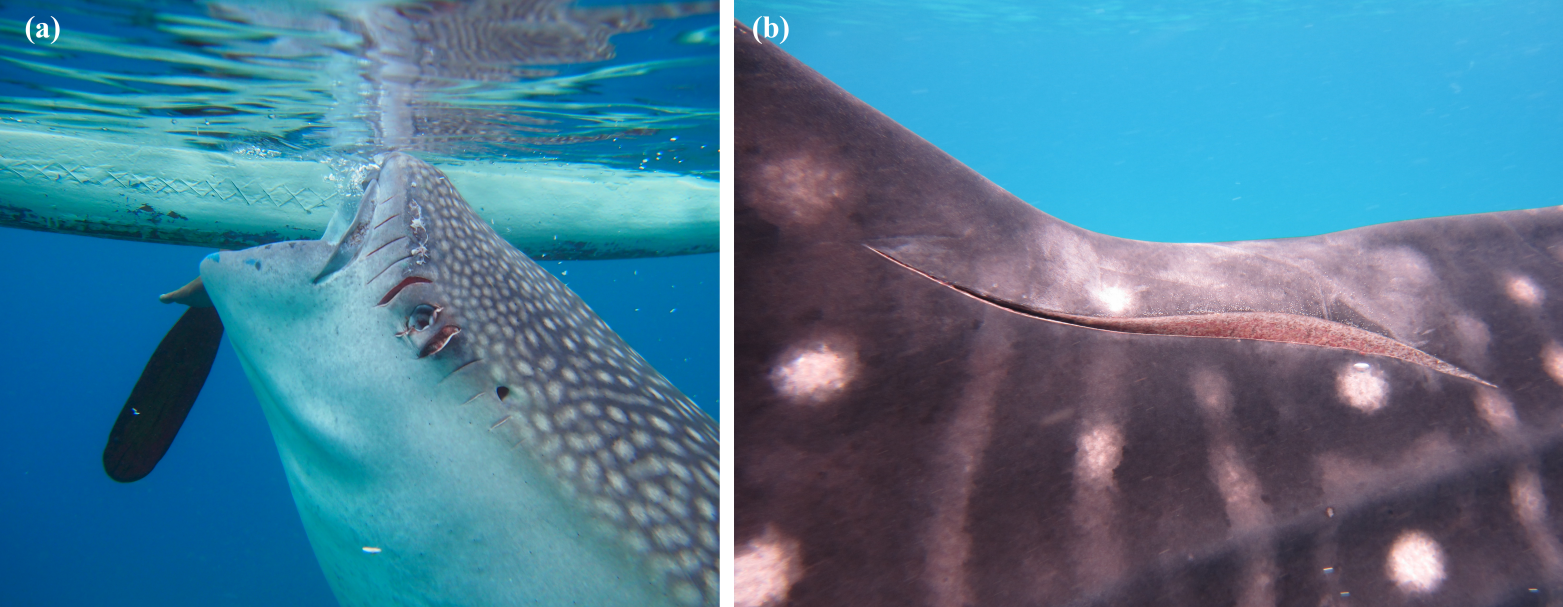
Examples of scars observed on *R. typus* from collision with small (A) and large (B) propellers.

Propeller scars were observed in 47% of the whale sharks (*n* = 158) probably derived from small outrigger boats with propeller diameter between 5 and 20 cm, or from larger commercial-vessel collisions (21–50 cm) (see Fig. 3). Scars were also used to aid individual identification.

### Presence

**Figure 4:**
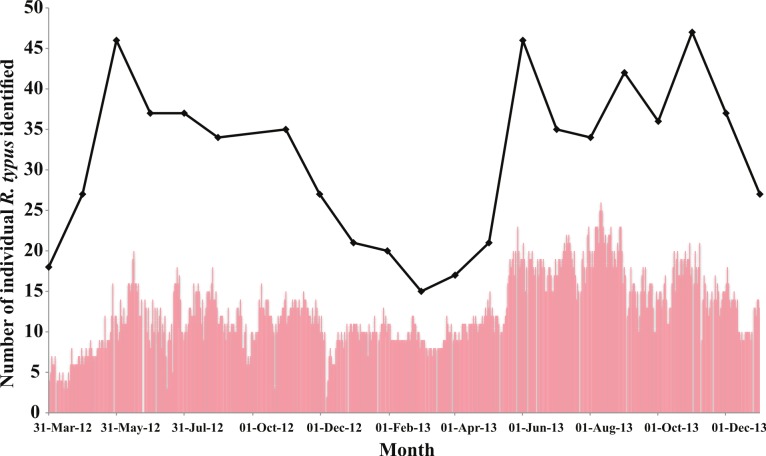
Presence of *R. typus* in Oslob between Mar 2012 and Dec 2013. Bars indicate the number of individual *R. typus* present daily in the interaction area. The line shows the number of individuals identified throughout each month of study.

Throughout the 621 surveyed days, a mean of 12.7 (±4.3 S.D.) individual whale sharks were seen in the survey area daily, with a maximum presence of 26 individuals (Aug 10 2013). A minimum presence of 2 individuals was recorded on Dec 6 2012, the first day of survey after Typhoon Pablo (International code name “Bopha”) made landfall in Southern Cebu on the Dec 4 2012. Given the length of the study period, the number of new individuals present in the interaction area was analysed by month. Monthly variations in the number of individual whale sharks present on at least one day during each month appear to suggest some seasonality (Fig. 4). During the first month of the study (Apr 2012) 18 individuals were present in the interaction area. Forty-six individual *R. typus* were identified throughout the months of June 2012 and May 2013. The maximum number of *R. typus* identified in the study area was seen throughout October 2013, totalling 47 animals. In Contrast, a minimum of 15 individuals were present during February 2013. Linear regression analysis of moon irradiance (%) posed no significant difference on the daily presence of *R. typus* individuals in the study area (*r*^2^ = 0.0015; *P* = 0.33).

**Figure 5:**
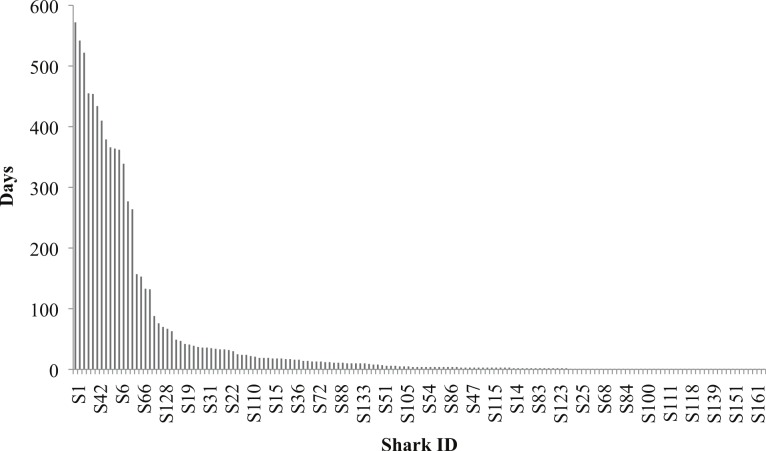
Histogram depicting the presence of each individual *R. typus* at the study site for the duration of the study (*n* = 621).

**Figure 6:**
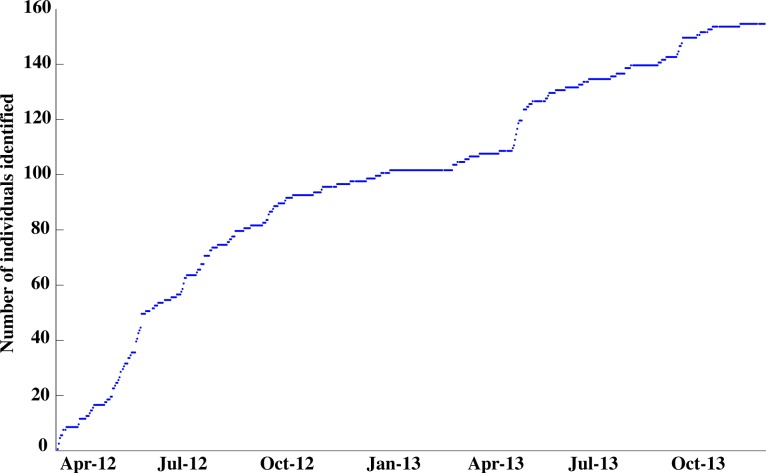
Discovery curve for newly identified *R. typus* in the interaction area for the duration of the study period.

Of the 158 individuals identified, 29% (45) were seen once, whereas 71% (113) were resighted (>1 day) in the interaction area. The mean presence of individuals in the interaction area was 49.9 days (±118.7 days S.D.). Twenty-three (14.6% *n* = 158) individuals were present for longer than the mean residency, and 12 individuals (7.6% *n* = 158) were identified in the interaction area at least 50% of survey days (*n* = 621). The maximum residency was observed on individual P-396 with a presence of 572 days, or 92% of survey days (Fig. 5). The discovery curve of newly identified *R. typus* over time also suggests seasonality as to when new individuals were identified in the study area, as indicated by a steeper climb during peak season (May–Jun, Fig. 6).

Through the use of *R. typus* unique spot pattern, “Wildbook for Whale Sharks” library, and the use of citizen scientists contributing to it, 11 individuals were matched in Southern Leyte, a province located 200 km East across the Bohol Sea. Using the same methods, two individuals were matched to pictures taken in Donsol (∼380 km North East), and another 11 individuals were matched in other regional diving tourism destinations including Malapascua Island (∼220 km North), Panglao Island (∼40 km East), and Moalboal (∼55 km North West). One individual was matched at both Malapascua Island and Donsol. These matches are summarised in Table 2.

**Table 2:** Summary table of *R. typus* matched across regional hotspots in the Philippines.

Shark ID	Match location	Source
COS-3	Pescador Island, Moalboal, Cebu	Citizen Scientist
COS-11	Moalboal, Cebu	Citizen Scientist
COS-12	Boljoon, Cebu	Citizen Scientist
COS-14	Limasawa, Southern Leyte	Authors
COS-35	San Ricardo, Southern Leyte	Authors
COS-46	Panglao Island, Bohol	Citizen Scientist
COS-47	Donsol, Sorsogon; Malapascua Island, Cebu	Citizen Scientist
COS-50	Pescador Island, Moalboal, Cebu	Citizen Scientist
COS-54	Panglao Island, Bohol	Citizen Scientist
COS-56	Pescador Island, Moalboal, Cebu	Citizen Scientist
COS-59	Moalboal, Cebu	Citizen Scientist
COS-90	San Ricardo, Southern Leyte	Authors
COS-105	Pintuyan, Southern Leyte	Citizen Scientist
COS-109	Donsol, Sorsogon	Wildbook for Whale Sharks
COS-125	San Ricardo, Southern Leyte	Authors
COS-126	San Ricardo, Southern Leyte	Authors
COS-129	Alona Beach, Bohol	Citizen Scientist
COS-137	Pescador Island, Moalboal, Cebu	Citizen Scientist
COS-138	Pescador Island, Moalboal, Cebu	Citizen Scientist
COS-141	San Ricardo, Southern Leyte	Authors
COS-148	San Ricardo, Southern Leyte	Authors
COS-155	San Ricardo, Southern Leyte	Authors
COS-156	San Ricardo, Southern Leyte	Authors
COS-160	San Ricardo, Southern Leyte	Authors

**Notes.**

Where Citizen Scientist relates to data shared by members of the public via direct collaboration, or via the Internet.

### Provisioned and non-provisioned individuals

Provisioned individuals (*n* = 54) had a mean ± S.D. estimated *L_T_* of 5.1 m (±1.3 m). Size was estimated for 87 (*n* = 104) non-provisioned whale sharks with a mean ± S.D. estimated *L_T_* 5.7 m (±1.3 m), showing a significant difference in size between the two groups (*WMW*, *P* = 0.0163). Provisioned whale sharks were seen between 2 and 572 days (*n* = 621) inside the interaction area (mean 135.6 ± 173.7 days S.D.), contrasting with non-provisioned individuals who were seen present between 1 and 63 non-consecutive days (mean 5.4 ± 9.1 days S.D.) (*WMW*, *P* = 2.2^e−16^). Moon irradiance had no significance on the presence of provisioned or non-provisioned individuals at the study site as explained by linear regression (*r*^2^ = 0.0006, *P* = 0.54; *r*^2^ = 0.0033, *P* = 0.15 respectively). Sex was determined for provisioned individuals with 49 males (91%, *n* = 54) and 5 females (9%, *n* = 54), and for non-provisioned individuals with 80 males (77%, *n* = 104), 14 females (13%, *n* = 104) and 10 of undetermined sex (10%, *n* = 104). Both groups were significantly male biased (*χ*^2^ = 19.6, *P* = 9.63e−06, and *χ*^2^ = 24.8, *P* = 6.20e−07, respectively). Propeller scars were observed on 51% of provisioned individuals, and on 44% of non-provisioned individuals, showing no significance (*χ*^2^ = 0.13, *P* = 0.72) relative to the overall population scarring (47%).

### Residency and lagged identification rate

The LIR and residency models showed that provisioned *R. typus* had a mean residency of 44.9 ± 20.6 S.E. (95% CI [38.5–113.4]) days, contrasting with a mean of 22.4 ± 8.9 S.E. (95% CI [6.0–36.2]) days on non-provisioned individuals. The mean time spent outside the study area was 22.6 ± 12.4 S.E. (95% CI [0.0–45.2]) days for provisioned individuals, and 94.7 ± 133.6 S.E. (95% CI [14.6–447.5]) days for non-provisioned individuals. The mean permanent emigration and mortality rate, where the animal is considered to have left the population, was modelled at 0.00031 ± 0.00022 S.E. (95% CI [−0.000084–0.000823]), and 0.003023 ± 0.001103 S.E. (95% CI [0.001437–0.006185]) for provisioned and non-provisioned whale sharks respectively.

**Figure 7:**
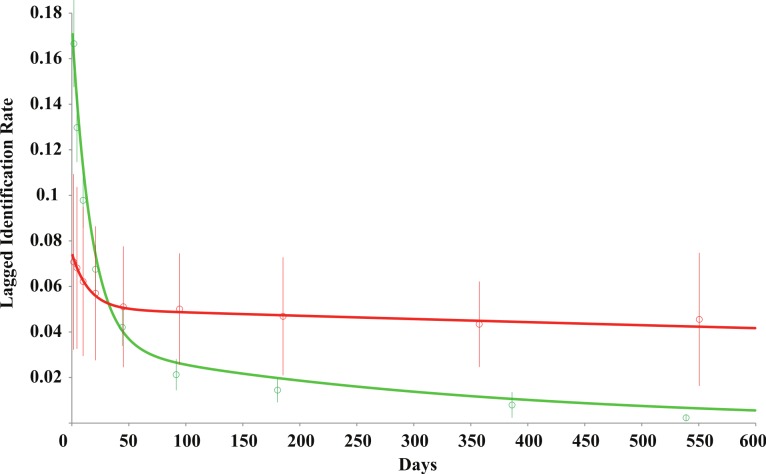
Lagged identification rate (LIR) for provisioned (red) and non-provisioned (green) *R. typus* at Oslob over increasing time periods. Modelled from fitted emigration + reimmigration + mortality rate (mean ± S.E.) (see Table 1).

The LIR was modelled for both datasets. The LIR for non-provisioned individuals showed a fast decrease from 1 to mean 93.3 days, after which the probability of resighting an individual decreased and stayed above zero for the remainder of the study (Fig. 7). Interestingly, there is a slight increased LIR at mean 379.2 days, potentially highlighting yearly seasonality. In contrast, LIR for provisioned individuals also showed a steep decrease from 1 to mean 94.9 days, however, the decrease in LIR over time was steady with only a 9.7% decrease from mean 47.4 to 554.7 days (Fig. 7). The LIR after 600 days of study period was 0.041707 and 0.005578 for provisioned and non-provisioned animals respectively, highlighting an almost 10-fold probability of resighting a provisioned whale shark than a non-provisioned one.

## Discussion

This is the first description of *R. typus* at a provisioning site in Southern Cebu, and the first publication describing the population structure of the species in the Philippines. A total of 158 individuals were identified, of which 82% were male, following the population structure of most sites across the globe, which is primarily juvenile male biased (Rowat & Brooks, 2012). The size of individuals ranged from 2.5 to 9.0 m *L_T_*, with only four males appearing to be sexually mature. The maturity of females was impossible to determine, though it has been suggested that it occurs >9.0 m, larger than the biggest female identified in Oslob (8.0 m) (Rowat & Brooks, 2012). The mean size of the animals (5.5 m) was similar to that of previously described aggregations from Ningaloo Reef, Baja California and the Maldives (Nelson & Eckert, 2007; Bradshaw et al., 2008; Riley et al., 2010), and slightly smaller than that described at Honduras, Gulf of Mexico, Qatar and the Seychelles (Graham & Roberts, 2007; de la Parra Venegas et al., 2011; Rowat et al., 2011; Robinson et al., 2013; Fox et al., 2013). While there were no individuals <2.0 m *L_T_* identified, three *R. typus* <2.0 m *L_T_* were previously reported (46 cm, 64 cm & 140 cm respectively) in Sorsogon Province near Donsol by Aca & Schmidt (2011), two of which were neonates, as they were smaller than the largest foetus described by Joung et al. (1996). The relative proximity of Oslob to this possible pupping ground and the connectivity established through photo-ID (Table 2) is a reminder of the importance of the area for the ecology of *R. typus*. This is however contrasted with the scarcity of sexually mature individuals, with only four males identified throughout the study, the largest of which was 9.0 m *L_T_*.

The use of photogrammetry as a non-invasive means to measure *R. typus*, and validate researchers’ estimated lengths, is an excellent approach. The results obtained for 20 *R. typus*, were only 0.2 m (±0.7 m) off those visually estimated lengths, indicating that researchers’ estimates are a good approximation of the real sizes. The relationship found was less significant (*r*^2^ = 0.83) than that obtained by Rohner et al. (2011), and can be attributed to using estimated lengths as opposed to total-length measurements with laser photogrammetry. However, it shows the important role this technique can play in improving accuracy on size estimation.

Propeller scars were observed in 47% of individuals. The fact that *R. typus* spends a significant amount of time at or near the surface makes it more vulnerable to vessel collision (Norman, 2002; Graham, Roberts & Smart, 2006; Rowat & Gore, 2007; Speed et al., 2008; Brunnschweiler et al., 2009; Brunnschweiler & Sims, 2011). In the Central Visayas region, where the study was conducted, small coastal villages use small motorized vessels with 5–20 cm diameter propellers to fish in near-shore waters, areas where *R. typus* is likely to visit for foraging. The percentage of individuals with propeller cuts within the Oslob population was higher than that recorded in Isla Holbox, Mexico (25%) (Ramírez-Macías et al., 2012). However, findings by Speed et al. (2008) did note that scarred individuals returned to the same aggregation, suggesting that propeller cuts do not necessarily impact habitat use. This was further supported by our observations in which individuals with fresh propeller cuts would return to the interaction area repeatedly. Given the high occurrence of propeller scars, propeller guards were highly recommended for operators visiting the study area, unfortunately none have been implemented yet.

Twenty-four individuals (15%, *n* = 158) were matched through Photo-ID and the use of their unique spot patterns, in other national hotspots, highlighting *R. typus* migratory nature as previously reported from both telemetry and Photo-ID studies (Eckert & Stewart, 2001; Eckert et al., 2002; Wilson et al., 2006; Graham & Roberts, 2007; Brunnschweiler et al., 2009; Brunnschweiler & Sims, 2011; McKinney et al., 2013). It also underlines the species’ large range, and the potential risk that the behavioural change induced by the provisioning poses to such migratory nature. To date, 650 individuals have been identified on “Wildbook for Whale Sharks” in the Philippines, meaning that Oslob potentially hosts 24% of the country’s population of *R. typus*.

The results of the present study show a significant difference in residency patterns between provisioned and non-provisioned individuals suggesting behavioural modification. This aggregation site is different to any other whale shark site previously identified and described because of the nature of the interaction, where the animals are attracted to the area with food. While a scientific description of provisioning *R. typus* has been lacking, a similar conditioning has been happening in Teluk Cenderawasih National Park, West Papua, Indonesia, but no data are yet available for comparison (Tania et al., 2013). Provisioning is more commonly used to attract apex predators where their presence is otherwise unreliable and unpredictable. Contrastingly, whale shark tourism interactions are based on their natural and reliable seasonal appearances at feeding sites (Rowat & Brooks, 2012).

The analyses showed no significant difference in sex and presence of propeller scars, between provisioned and non-provisioned individuals, however there was a significant significance in their residency times (*WMW*, *P* = 2.2^e−16^). Fifty-four individuals were recorded doing so, 12 of which were present in the study area over 50% of survey days (*n* = 621). Site fidelity of this magnitude has never been described in *R. typus*, where individuals are resighted year-round for a prolonged period of time in the same relatively small area, highlighting the potential conditioning the provisioning activities can have on these whale sharks as clearly exemplified by individual P-396 who was seen on 572 days (92% of surveyed days). Provisioned individuals (5.1 ± 1.3 m) were significantly smaller than non-provisioned individuals (5.7 ± 1.3 m), but could arguably be attributed to the smaller sample size. Figure 4 depicts the number of individuals present daily, and monthly, highlighting an increase of daily individuals over time (*r*^2^ = 0.29, *P* < 0.05). This could be recruitment of new non-provisioned individuals that learn how to feed from the feeder boats or individuals attracted to the site by the food pulse. Non-provisioned individuals were observed swimming slowly and showing curiosity, approaching snorkelers and boats alike, possibly attracted by the large amount of food dispersed in the water during the provisioning activities. Forty-five individuals were only seen once (28%, *n* = 158), indicating that the species probably migrate through the area.

The residency of *R. typus* was modelled to understand their presence in Oslob and contrast to other areas. This model described the provisioned individuals to have a residency time twice as long (44.9 ± 20.6 S.E. days) as non-provisioned ones (22.4 ± 8.9 S.E. days). With a dataset spanning through 10 years, Fox et al. (2013) modelled a mean residency of 11.76 ± 4.54 days at Utila, and through mark-recapture modelling, residency by *R. typus* at Ningaloo Reef was estimated at 33 days (Holmberg, Norman & Arzoumanian, 2009). The latter is higher than non-provisioned individuals in Oslob, though this can be attributed to the fact that this study was conducted inside a small interaction area, whereas at Ningaloo animals are sighted throughout a much larger area. Unfortunately few data are available for comparison using the same methods used in this study on estimating residency. Similarly, the LIR results show that after 621 days the probability of resighting a non-provisioned individual is only 13% of that of a provisioned individual. Results using the same statistical approach, from Ramírez-Macías, Vázquez-Haikin & Vázquez-Juárez (2012), showed a decline in LIR between 3–30 days at one location, and 3–60 days at their second study site, the latter suggesting slightly longer residency time. In Honduras, the LIR also declined sharply after 16–31 days (Fox et al., 2013). The results from these sites are similar to those found here, though the LIR of provisioned individuals stayed at a relatively high level over the duration of the study period. The mortality rate and permanent emigration modelled for non-provisioned individuals was also ∼10-fold that of provisioned individuals, suggesting they are much likelier to leave the study area. These differences in both residency and probability of being resighted at one particular site over time suggest behavioural modification induced by the provisioning activities.

## Conclusion

The population of *R. typus* visiting the waters of Oslob follow a similar structure in terms of size and sex distribution to many populations around the globe. The residency in this study area is however, considerably higher than that previously described, as is the lagged identification rate modelled though maximum likelihood methods for provisioned individuals. The short and long term impacts of such prolonged residency in one area for a highly migratory species like the whale shark needs further investigation. The results presented here underline behavioural differences between provisioned and non-provisioned individuals but caution is advised extrapolating the definition of provisioned animals beyond the scope of this study. Approximately 34% of the population was categorised as being provisioned, and are therefore potentially affected by this human interference with their natural ecology. Further work will focus on the dietary differences in provisioned and non-provisioned individuals, as well as other behavioural changes not directly observed or measured through residency, preferably through the use of telemetry technology. The environmental impact of the provisioning activities on the local ecosystems beyond the species under direct study will also be explored. This coupled with the socio-economical aspects of the provisioning activities will be pursued to delineate the limits of acceptable change and suggest a management plan to the Philippine National Government.
